# Complete genome sequence of *Parvimonas parva*: first isolate of a human clinical specimen from Japan

**DOI:** 10.1128/mra.01252-24

**Published:** 2025-02-07

**Authors:** Masahiro Hayashi, Jun Yonetamari, Yoshinori Muto, Kaori Tanaka

**Affiliations:** 1 Institute for Glyco-core Research iGCORE, Gifu University, Gifu City, Gifu, Japan; 2 Division of Anaerobe Research, Life Science Research Center, Gifu University, Gifu City, Gifu, Japan; 3 Gifu University Center for Conservation of Microbial Genetic Resource, Gifu City, Gifu, Japan; 4 Division of Clinical Laboratory, Gifu University Hospital 476117 , Gifu City, Gifu, Japan; 5 United Graduate School of Drug Discovery and Medical Information Sciences, Gifu University, Gifu City, Gifu, Japan; Rochester Institute of Technology, Rochester, New York, USA

**Keywords:** *Parvimonas*, genomes, anaerobes

## Abstract

*Parvimonas parva* is a bacterium belonging to the *Peptoniphilaceae* family, which was first classified in 2021. Herein, we report the complete genome sequence of *P. parva* (GAI15033) isolated from a clinical sample in Japan. The genome comprises a 1,447,534 bp circular chromosome.

## ANNOUNCEMENT

*Parvimonas parva* is a gram-positive, strictly anaerobic bacterium belonging to the *Peptoniphilaceae* family, registered in 2021 (1). The strain in this study (GAI15033) was isolated from the urine sample of an elderly woman with a tumor in the urogenital system, anaerobically cultured, and submitted to our lab as a suspected *Parvimonas* strain for further identification in 2015. The strain was cultured on Brucella agar with hemin and vitamin K supplemented with 5% laked sheep blood at 37°C for 48 h under anaerobic conditions, and subjected to API 20A and RapidID 32A tests (bioMérieux Japan Ltd., Tokyo, Japan); analysis confirmed identification at the genus level (*Parvimonas spp*.). The strain was stored at −80°C after several passages.

The strain was cultured under conditions used for isolation. After incubation, colonies were scraped, suspended in Tris-EDTA buffer, and pelleted through centrifugation. Genomic DNA was extracted from the pellet using MonoFas (ANIMOS, Tokyo, Japan).

The 16s rRNA gene was sequenced after PCR amplification using primers 16S-8F (AGAGTTTGATCMTGGCTCAG) and 16S-1492R (GGYTACCTTGTTACGACTT). BLASTN analysis (2) of PCR-amplified full-length 16S rRNA gene identified the strain as *Parvimonas parva* S3374 T (Acc.no. MT982357) with 99.93% similarity.

The entire genome was sequenced using a combination of long-read nanopore sequencing (Oxford Nanopore Technologies [ONT], Tokyo, Japan) and short-read sequencing using DNBSEQ (MGITech Co., Ltd., Shenzhen, China) as previously described (3, 4). For long-read sequencing, a library was constructed using a ligation sequencing kit (SQK-LSK-109; ONT) without shearing. Small DNA fragments were removed using a Short Read Eliminator kit (Pacific Bioscience of California, Inc, USA). Sequencing was performed using a GridION X5 system (ONT) on a FLO-MIN106 flow cell. Long-read sequencing data were base-called using Guppy v.5.0.12 (high accuracy mode). Raw reads were trimmed and quality filtered using NanoFilt v.2.7.1 (5) with parameters “-l 1000 -q 10 --headcrop 50.” For short-read sequencing, MGIEasy FS PCR-Free DNA library prep set (MGITech) was used for library construction. 2 × 150 bp paired-end sequencing was performed using the DNBSEQ-G400 platform (MGI Tech). Raw sequencing reads were processed using fastp v.0.20.1 (6) with parameters “-q 30 n 20 t 1 T 1.” Quality of short reads and long reads were assessed using fastp v.0.20.1 (6), and NanoPlot 1.32.1 (7), respectively. High-quality short reads (over 91% of bases > Q30 averaged) and long reads (mean read quality of 13.2) were assembled using Unicycler v.0.4.8 (8) with default settings. The assembly was rotated to start with the forward strand’s *dnaA* gene. The assembled contig graph was visualized using Bandage v.0.8.1 (9), and assembled genomic data integrity was confirmed using blobtools v.1.1 (10).

Genome information is summarized in Table 1. Quality assessment and genome statistics were computed using QUAST (v5.2.0) (7) and CheckM (v1.2.2) (11). The genome assembled in this study was 100% complete with 0.0% contamination. DDBJ Fast Annotation and Submission Tool (12) analyzed 1392 coding sequences, 10 ribosomal RNAs, and 41 transfer RNAs in total. Species identity was confirmed with an average nucleotide identity of 97.98% against *P. parva* type strain S3374 (ASM1655216v1) using GTDB-tK 2.3.2 (13).

**TABLE 1:** Information regarding the complete genome sequence of a *Parvimonas parva* strain isolated from clinical isolate in Japan

	Strain name
Parameter	*Parvimonas parva* GAI15033
DNBSEQ sequencing^a^	
No. of reads	22,982,810
Size (kb)	3,447,422
Avg coverage (x)	2,382
DRA accession no.	DRR596924
ONT sequencing^a^	
No. of reads	579,389
Size (kb)	2,444,077
Avg read length (bp)	4,218
Avg coverage (x)	1,688
N50	1447534
DRA accession no.	DRR596923
Assembly	
Assembly N50 (bp)^b^	
Estimated genome completeness (%)^c^	100.0
Estimated genome contamination (%)^c^	0.0
Genome structure	one chromosome
DDBJ/GenBank accession no.	AP038371
Genome size (bp)	1,447,534
GC content (%)	
(chromosome/ plasmid name)	28.7
No. of coding sequences^d^	1,392
Number of rRNAs^d^	10
Number of tRNAs^d^	41
Number of CRISPRs^d^	1

^a^ DRA, DDBJ Sequence Read Archive.

^b^ Determined with QUAST.

^c^ Determined with CheckM.

^d^ DFAST, DDBJ Fast Annotation and Submission Tool.
